# Supplementary material for: Plant data visualisation using network graphs
Source: PeerJ. 2018 Aug 31;6:e5579. doi: 10.7717/peerj.5579 (PMC6120445; doi:10.7717/peerj.5579)
Supplement: Supplemental Information 1 — The detailed manual on how to use the PlantViz. [file peerj-06-5579-s001.pdf]

Manual on How to Use PlantViz

To use the PlantViz, click on the **PlantViz** tab as shown in Fig. 1.

PlantViz provides a graphical user interface that enable users to query the POUM and a graphical viewer to display the results of the query in a form of network graph. The relationships between the data were shown in visualisation form that enables the users to infer the knowledge and correlation between the data easily.

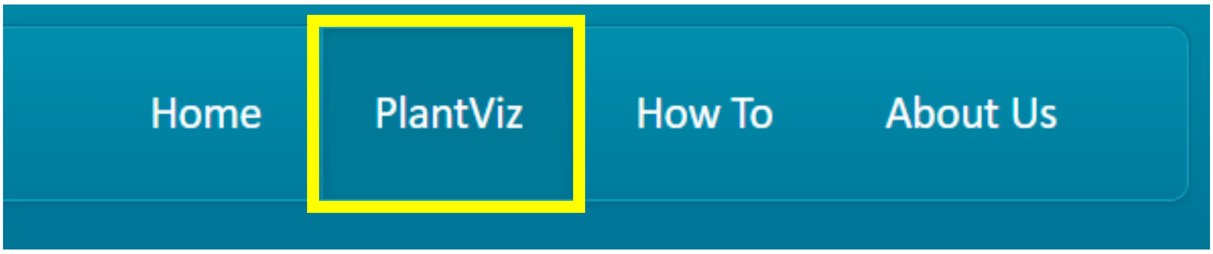

Figure 1

Searching using Query Tool

Searching in PlantViz is based on the four query parameter options namely 'Scientific Name', 'Family Name', 'Location', and 'Water Usage' (see Fig. 2).

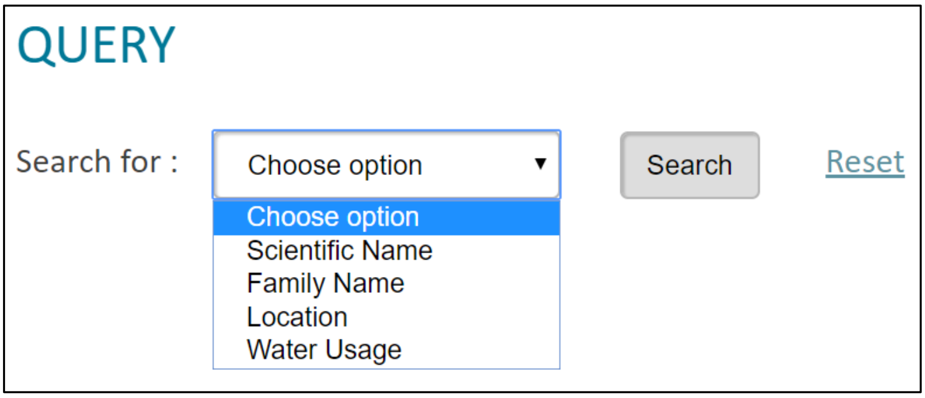

Figure 2

1. To start, users are required to select one of these query options at a time. Once the option is selected, users are required to key in a keyword or select relevant parameter from the given options in drop down list (see Figs. 3-6).

Query parameter: Scientific Name

- The keyword must start with an uppercase letter for the genus name. Example: "**I**xora javanica"
- The words must be separated with a single space. Example: "Lawsonia**<space>**inermis"
- There should be no extra space at the beginning or at the end of keyword.

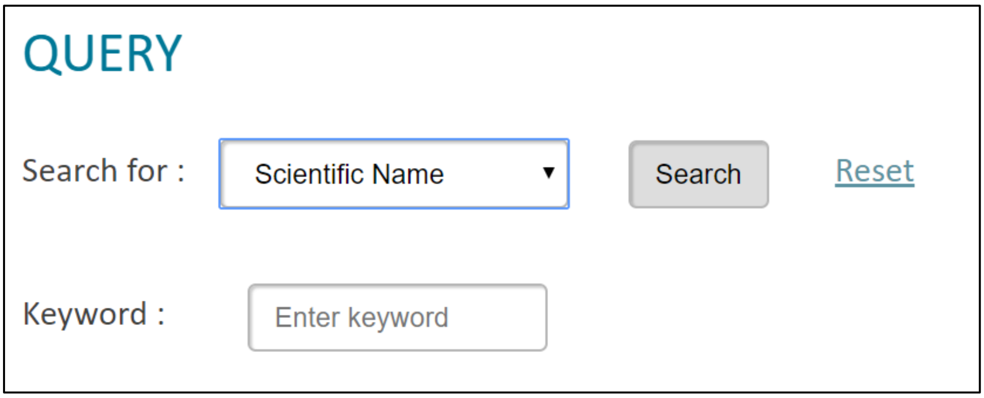

Figure 3

Query parameter: Family Name

- The keyword must start with an uppercase letter. For example: “**A**canthaceae”
- There should be no extra space at the beginning or at the end of keyword.

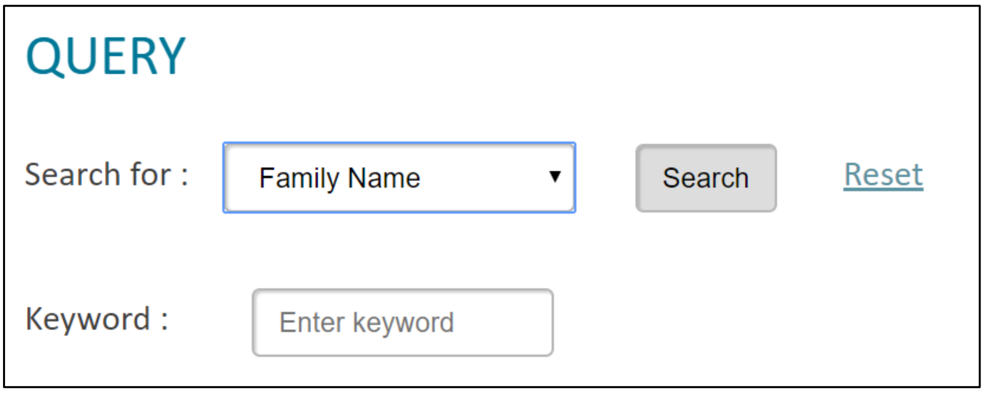

Figure 4

Query parameter: Location

- You can only select one from the given options.

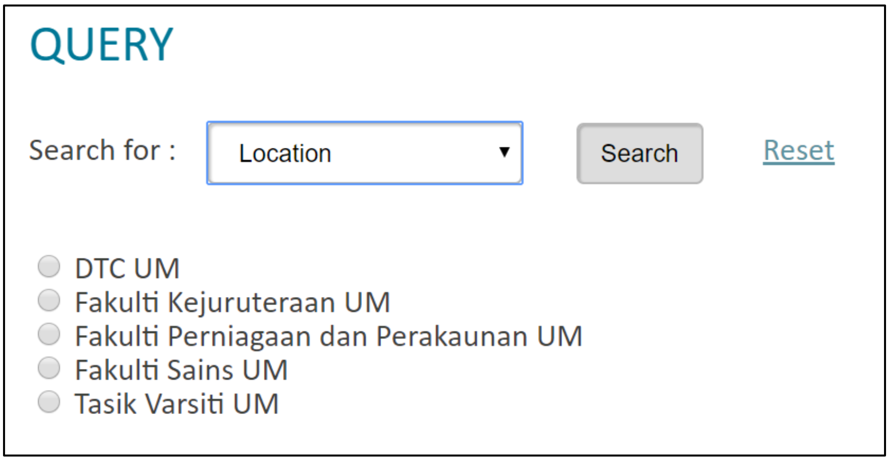

Figure 5

Query parameter: Water Usage

- You can only select one from the given options.

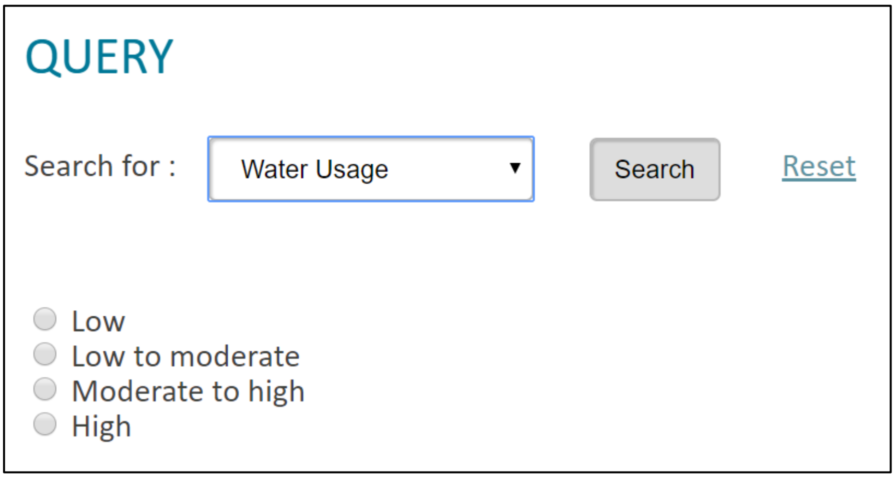

Figure 6

2. Once the query parameter option is selected and keyword is entered, or an option in drop down list is selected, click on the **Search** button to start the searching.
3. To start over, click on the **Reset** button.
4. Please do not click on **Back** button to start a new query for searching.

Visualization Features in Graphical Viewer

PlantViz presents the retrieved result of a query in a graphical form to allow user to see the relationships between the data instead of reading it. Users are able to interact directly with the presented data by using the visualization features provided in PlantViz as well as basic features which include **zoom in**, **zoom out**, and **draggable**.

1. View label of the nodes

The purpose of this feature is to allow user to view the label of the nodes (see Fig. 7).

1. Hover the cursor on the • node.
2. Hold the cursor until the label on the node is appeared.
3. If the label does not appear, click on the • node and hover it again.

   Note: The other nodes may also have labels

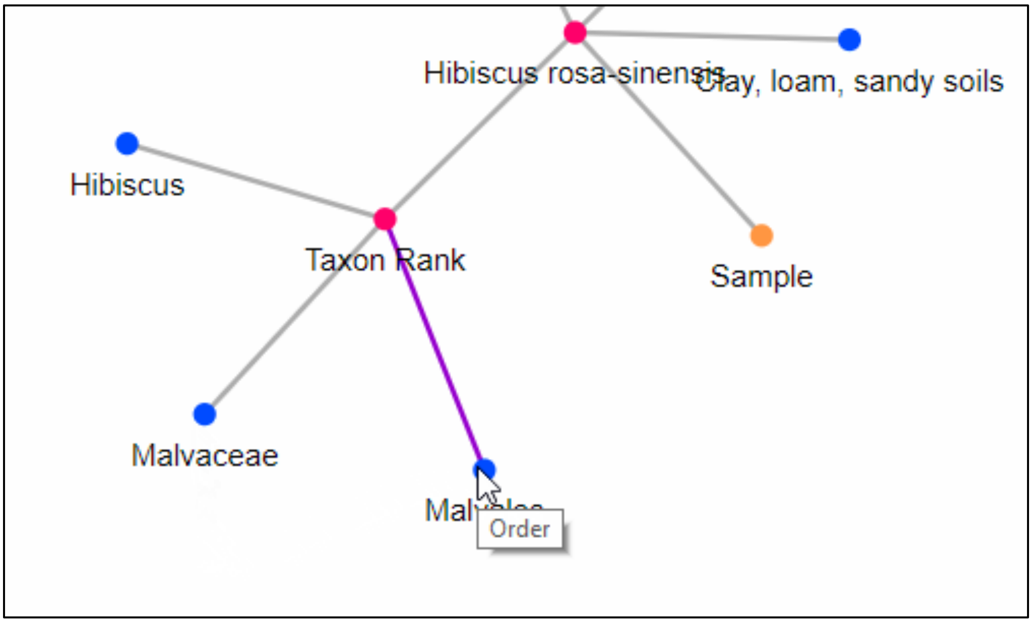

Figure 7

2. Highlight nodes

The purpose of this feature is to show relationships between the data (see Fig. 8).

1. Hover the cursor on any node.
2. The links that connected the nodes will be highlighted in purple colour.

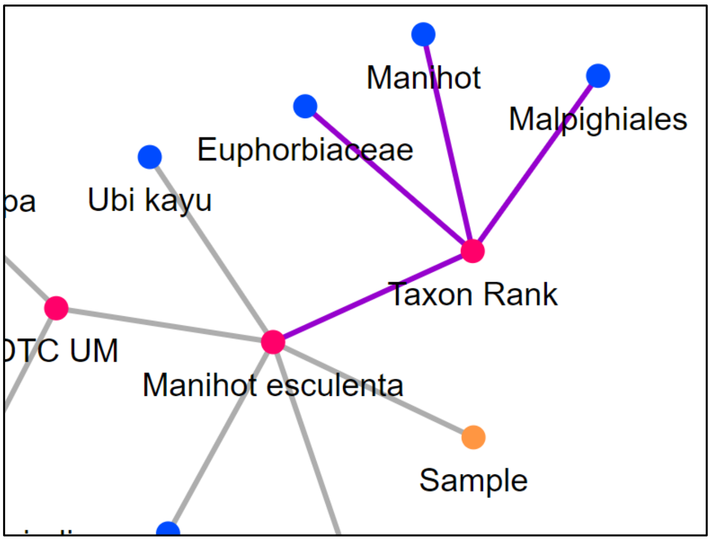

Figure 8

3. Expand and shrink the network graph

The purpose of this feature is to avoid cluttered nodes in the network graph. It also helps users to focus on a part of the presented data.

1. To shrink the graph, double click on the • node (see Fig. 9)

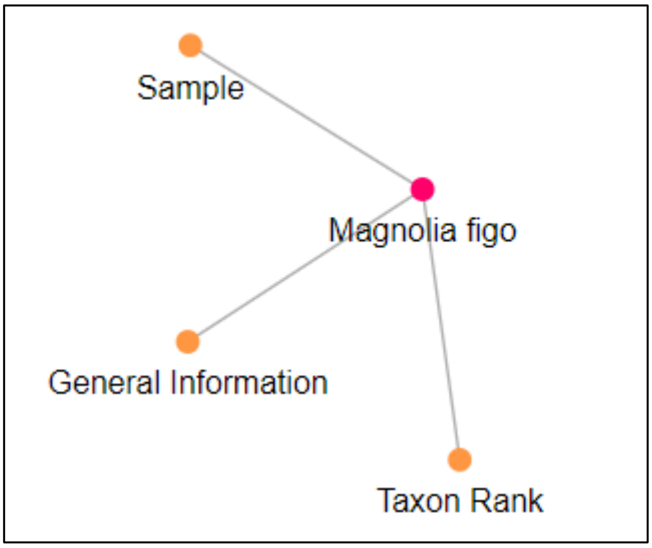

Figure 9

2. To expand the network graph, double click on the • node (see Fig. 10).

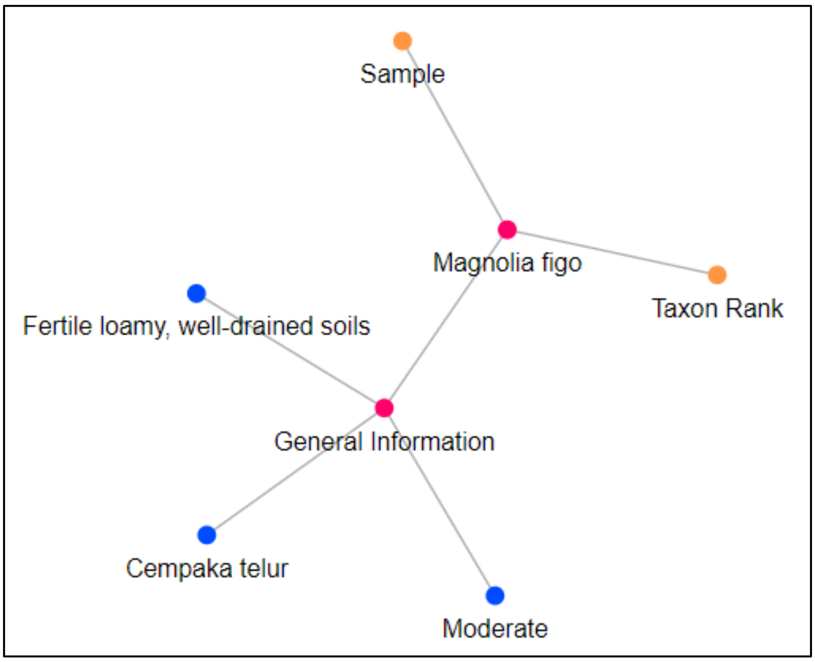

Figure 10

4. View the thumbnail images of plant sample

The purpose of this feature is to view the thumbnail image(s) of some plant sample(s) (see Fig. 11).

1. Hover the cursor on the node with the sample ID.
2. A popup with thumbnail image(s) will be appeared.

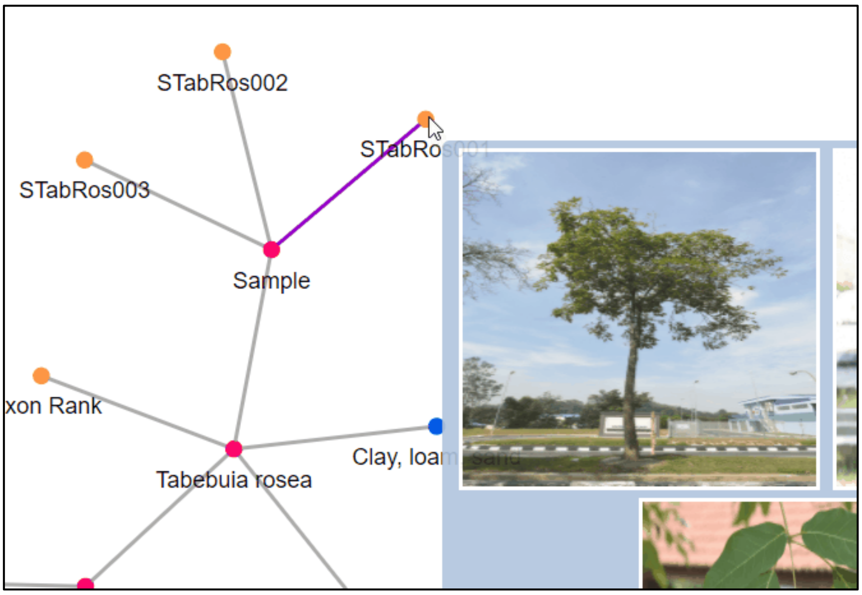

Figure 11

5. **View full information of selected plant sample**

The purpose of this feature is to view full information of selected plant sample (see Fig. 12).

1. Hover the cursor on the **Sample** node.
2. Click on the **Sample details** node and users will be directed to a new page containing full information of the selected plant sample.

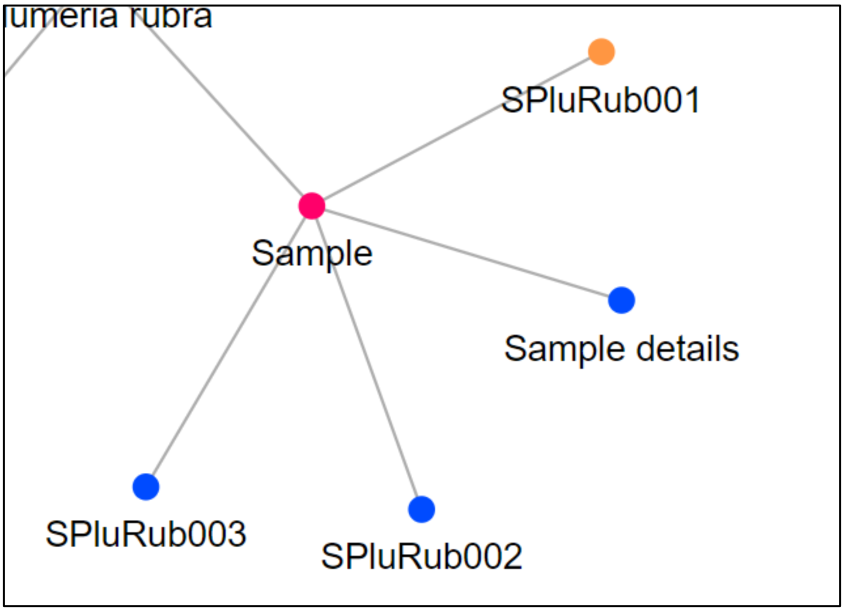

Figure 12
