# Supplementary material for: Plant data visualisation using network graphs
Source: PeerJ. 2018 Aug 31;6:e5579. doi: 10.7717/peerj.5579 (PMC6120445; doi:10.7717/peerj.5579)
Supplement: Supplemental Information 2 — Standard vocabularies representing the classes and properties used in POUM. [file peerj-06-5579-s002.docx]

Table S1: Classes and properties of POUM

| **Terms** | **Description** |
| --- | --- |
| **Main Class** | |
| PlantSample | Record of the plant sample. |
| Species | Record of the scientific name of the plant sample. |
| Description | General characteristics of each part of a plant species (tree, bark, fruit, flower, leaf). |
| TaxonRank | Taxonomical information of scientific names. |
| ImgProperties | Details of plant images. |
| Habitat | Details of plant habitats. |
| Distribution | Location of plant samples. |
| Parts | Description of the parts of plants and plant samples (tree, bark, fruit, flower, leaf). |
| **Sub Class** | |
| Leaf | Description of the leaf of the plant and plant sample. |
| Stem | Description of the stem of the plant and plant sample. |
| Fruit | Description of the fruit of the plant and plant sample. |
| Flower | Description of the flower of the plant and plant sample. |
| Whole | Description of the tree of the plant and plant sample. |
| **Object Property** | |
| consistOf | Components of the plant samples. |
| isSpecies | Details of the scientific name of the plant samples. |
| hasDesc | Description of a plant species. |
| of | Components of the plants. |
| hasImg | Properties of the plant sample’s images. |
| livesIn | Location of the plant sample. |
| hasRank | Details of the hierarchy of a scientific name. |
| hasSample | Information of collected samples of the plants. |
| isBelongTo | Refers to level of a rank to the upper level of the taxonomical hierarchy. |
| hasSpecies | Refers to the species level of the taxonomical hierarchy. |
| hasClass | Refers to the class level of the taxonomical hierarchy. |
| hasDivision | Refers to the division level of the taxonomical hierarchy. |
| hasFamily | Refers to the family level of the taxonomical hierarchy. |
| hasGenus | Refers to the genus level of the taxonomical hierarchy. |
| hasHabitat | Habitat of the plant species. |
| isA | Components of the plants’ parts. |
| isCitedFrom | Reference for where the data of plants are obtained |
| **Data Property** | |
| sampleId | Unique identifier for plant samples. |
| plantName | Common name for plants. |
| scientificName | Scientific name of plant species. |
| scientificNameAuthorship | Full name of the author. |
| yearPublished | Year when the scientific name is published. |
| Class | Class of the plant’s scientific name. |
| Division | Division of the plant’s scientific name. |
| Family | Family of the plant’s scientific name. |
| Genus | Genus of the plant’s scientific name. |
| Kingdom | Kingdom of the plant’s scientific name. |
| Order | Order of the plant’s scientific name. |
| taxonRank | Rank of each level in taxonomical hierarchy. |
| Species | Species of the plant’s scientific name. |
| fileFormat | File format of the image. |
| imgSize | Memory size of the image. |
| imgObject | Object of the image. |
| imgPath | Location of the image saved in folder. |
| habitatType | Condition of the habitat’s environment. |
| typeOfSoil | Type of the habitat’s soil. |
| waterUsage | Amount of water use for plants. |
| geoSpatialCoordinates | Decimal latitude(s) and longitude(s) of places of origin of the material in the collection. |
| geoSpatialCoverage | Place names from which material in the collection originated from. |
| leafArrangement | Arrangement of leaf in a stem |
| leafBase | Type of leaf’s base. |
| leafMargin | Type of leaf’s edge. |
| leafShape | Type of leaf’s shape. |
| leafWidth | Width of a leaf. |
| leafLength | Length of a leaf. |
| leafSurface | Type of leaf’s surface. |
| leafTip | Type of leaf’s tip. |
| leafType | Type of a leaf (compound/single). |
| leafVenation | Type of leaf’s veins. |
| barkColor | Color of the bark. |
| barkSurface | Type of bark’s texture. |
| fruitColor | Color of a fruit. |
| flowerColor | Color of a flower. |
| infloresecenceType | Shape of a flower. |
| petalNum | Number of petals. |
| treeHeight | Height of a tree. |
