# Supplementary material for: Plant data visualisation using network graphs
Source: PeerJ. 2018 Aug 31;6:e5579. doi: 10.7717/peerj.5579 (PMC6120445; doi:10.7717/peerj.5579)
Supplement: Supplemental Information 3 — There are 43 species of 42 genera for trees in POUM. [file peerj-06-5579-s003.docx]

Table S2: List of family, genus, and species for tree

| **Family** | **Genus** | **Species** |
| --- | --- | --- |
| Anacardiaceae | Mangifera | *Mangifera indica* |
| Annonaceae | Polyalthia | *Polyalthia longifolia* |
| Apocynaceae | Alstonia | *Alstonia angustiloba* |
|  | Plumeria | *Plumeria rubra* |
| Bignoniaceae | Spathodea | *Spathodea campanulata* |
|  | Tabebuia | *Tabebuia rosea* |
| Calophyllaceae | Mesua | *Mesua ferrea* |
| Combretaceae | Bucida | *Bucida molinetii* |
|  | Terminalia | *Terminalia catappa* |
| Dipterocarpaceae | Dipterocarpus | *Dipterocarpus grandiflorus* |
|  | Dryobalanops | *Dryobalanops aromatica* |
|  | Hopea | *Hopea odorata* |
| Euphorbiaceae | Hura | *Hura crepitans* |
| Fabaceae | Acacia | *Acacia auriculiformis* |
|  | Adenanthera | *Adenanthera pavonina* |
|  | Albizia | *Albizia saman* |
|  | Bauhinia | *Bauhinia blakaena* |
|  | Cassia | *Cassia fistula* |
|  | Cynometra | *Cynometra malaccensis* |
|  | Delonix | *Delonix regia* |
|  | Erythrina | *Erythrina variegata* |
|  | Hymenaea | *Hymenaea courbaril* |
|  | Pterocarpus | *Pterocarpus indicus* |
|  | Saraca | *Saraca thaipingensis* |
|  | Senna | *Senna surattensis* |
| Gentianaceae | Fagreae | *Fagraea fragrans* |
| Lauraceae | Cinnamomum | *Cinnamomum iners* |
| Lecythidaceae | Barringtonia | *Barringtonia racemosa* |
| Lythraceae | Lagerstroemia | *Lagerstroemia floribunda* |
| Malvaceae | Sterculia | *Sterculia foetida* |
|  | Theobroma | *Theobroma cacao* |
| Meliaceae | Khaya | *Khaya senegalensis* |
|  | Swietenia | *Swietenia macrophylla* |
| Moraceae | Artocarpus | *Artocarpus integer* |
|  | Ficus | *Ficus microcarpa* |
| Myrtaceae | Eucalyptus | *Eucalyptus alba* |
|  | Melaleuca | *Melaleuca cajuputi* |
|  | Syzygium | *Syzygium aqueum*  *Syzygium campanulatum* |
|  | Tristaniopsis | *Tristaniopsis whiteana* |
| Sapindaceae | Filicium | *Filicium decipiens* |
| Sapotaceae | Mimusops | *Mimusops elengi* |
| Thymelaeaceae | Aquilaria | *Aquilaria malaccensis* |
