# Supplementary material for: Plant data visualisation using network graphs
Source: PeerJ. 2018 Aug 31;6:e5579. doi: 10.7717/peerj.5579 (PMC6120445; doi:10.7717/peerj.5579)
Supplement: Supplemental Information 4 — There are 31 species of 28 genera for shrubs in POUM. [file peerj-06-5579-s004.docx]

Table S3: List of family, genus, and species for shrub

| **Family** | **Genus** | **Species** |
| --- | --- | --- |
| Acanthaceae | Clinacanthus | *Clinacanthus nutans* |
|  | Graptophyllum | *Graptophyllum pictum* |
|  | Strobilanthes | *Strobilanthes crispa* |
| Apocynaceae | Allamanda | *Allamanda cathartica* |
|  | Tabernaemontana | *Tabernaemontana divaricata* |
| Araliaceae | Polyscias | *Polyscias balfouriana* |
| Asparagaceae | Dracaena | *Dracaena reflexa*  *Dracaena surculosa* |
| Dilleniaceae | Dillenia | *Dillenia suffruticosa* |
| Euphorbiaceae | Acalypha | *Acalypha siamensis*  *Acalypha wilkesiana* |
|  | Excoecaria | *Excoecaria cochinchinensis* |
|  | Manihot | *Manihot esculenta* |
| Hamamelidaceae | Loropetalum | *Loropetalum chinense* |
| Lythraceae | Lagerstroemia | *Lagerstroemia indica* |
|  | Lawsonia | *Lawsonia inermis* |
| Magnoliaceae | Magnolia | *Magnolia figo* |
| Malvaceae | Hibiscus | *Hibiscus rosa-sinensis* |
|  | Malvaviscus | *Malvaviscus arboreus* |
| Melastomataceae | Melastoma | *Melastoma malabathricum* |
|  | Tibouchina | *Tibouchina urvilleana* |
| Nyctaginaceae | Bougainvillea | *Bougainvillea spectabilis* |
| Phyllanthaceae | Phyllanthus | *Phyllanthus myrtifolius* |
|  | Sauropus | *Sauropus androgynus* |
| Rubiaceae | Ixora | *Ixora javanica* |
|  | Mussaenda | *Mussaenda erythrophylla*  *Mussaenda philippica* |
| Rutaceae | Murraya | *Murraya paniculata* |
| Solanaceae | Bruntelsia | *Brunfelsia calycina* |
| Verbenaceae | Duranta | *Duranta erecta* |
|  | Lantana | *Lantana camara* |
