# Supplementary figures and images for: Plant data visualisation using network graphs
Source: PeerJ. 2018 Aug 31;6:e5579. doi: 10.7717/peerj.5579 (PMC6120445; doi:10.7717/peerj.5579)

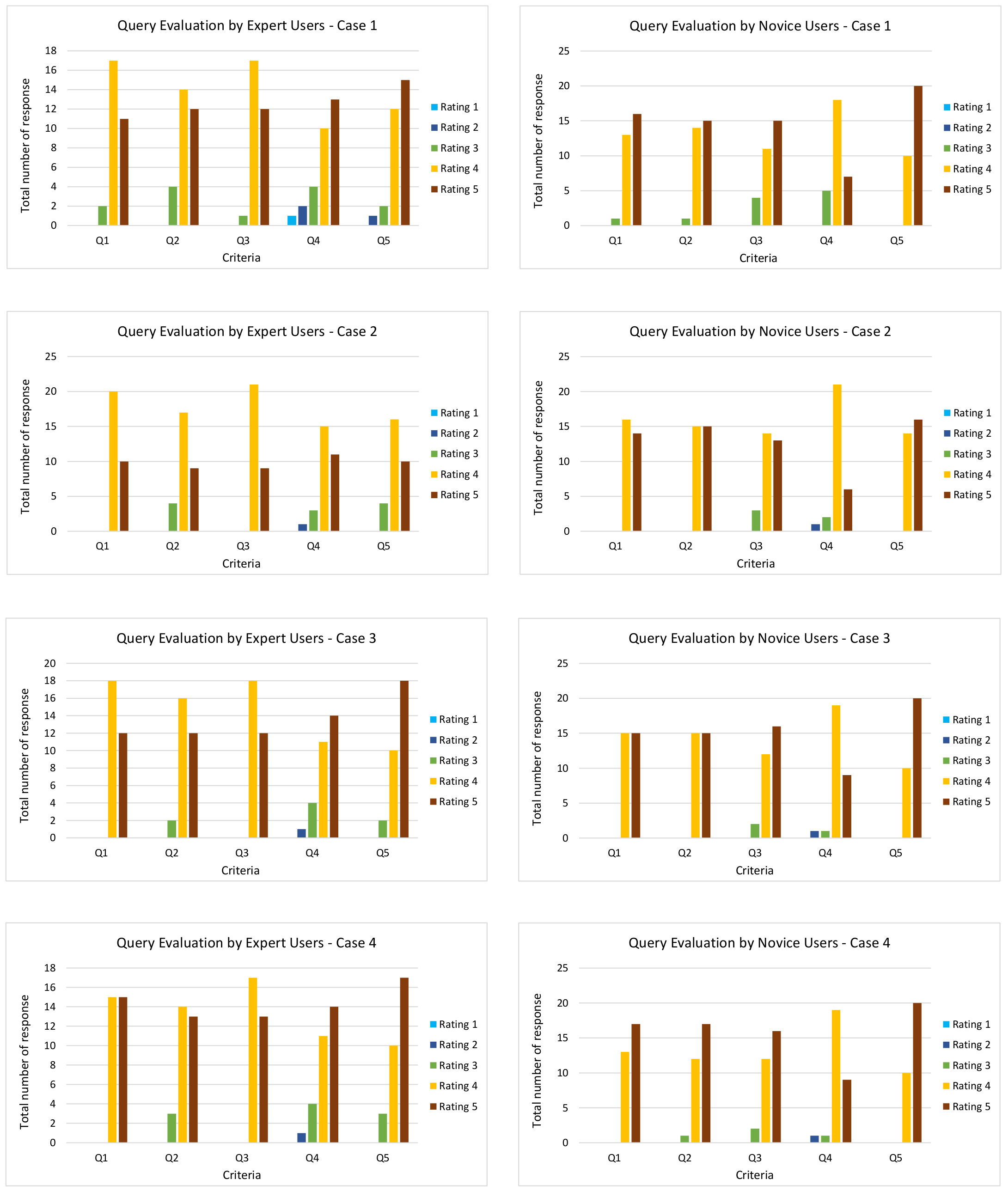

Supplement: Supplemental Information 5 — Complete analyses of query evaluation for all four cases. [file peerj-06-5579-s005.png]

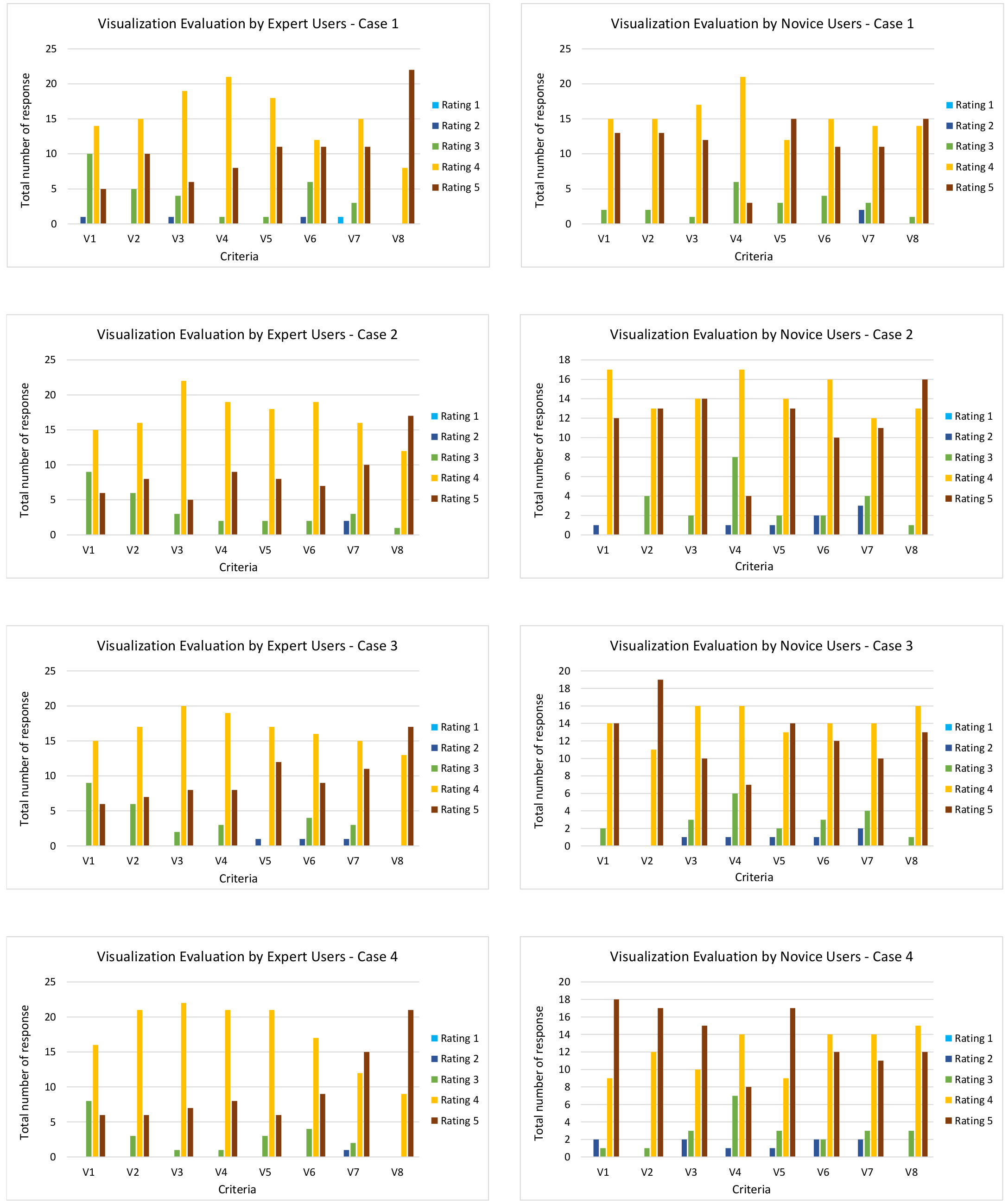

Supplement: Supplemental Information 6 — Complete analyses of visualisation evaluation for all four cases. [file peerj-06-5579-s006.png]
